# Supplementary material for: Universal symmetry-protected persistent spin textures in noncentrosymmetric crystals
Source: Nat Commun. 2025 Aug 27;16:7999. doi: 10.1038/s41467-025-63136-4 (PMC12391364; doi:10.1038/s41467-025-63136-4)
Supplement: Supplementary file 1 — Supplementary Information [file 41467_2025_63136_MOESM1_ESM.pdf]

Supplementary Information for "Universal symmetry-protected persistent spin textures in noncentrosymmetric crystals"

Berkay Kilic[1], Sergio Alvarruiz[1], Evgenii Barts[1,2], Bertjan van Dijk[1], Paolo Barone[3], and Jagoda Sławińska[1]

[1]Zernike Institute for Advanced Materials, University of Groningen, Nijenborgh 3, 9747 AG Groningen, The Netherlands
[2]Quantum Materials Theory, Italian Institute of Technology, Via Morego 30, 16163 Genoa, Italy
[3]CNR-SPIN Institute for Superconducting and other Innovative Materials and Devices, Area della Ricerca di Tor Vergata, Via del Fosso del Cavaliere 100, I-00133 Rome, Italy

Supplementary note 1

$\boldsymbol{k}\cdot\boldsymbol{p}$ perturbation theory within a group-theoretical approach

Within a one-particle approximation, the electronic properties of a crystalline system are described by a set of one-particle wave functions satisfying a self-consistent Schrödinger equation:

$$H\psi_{n\boldsymbol{k}} = \epsilon_n(\boldsymbol{k})\psi_{n\boldsymbol{k}}, \tag{1}$$

where the Hamiltonian can be decomposed as

$$H = H_{\mathrm{K}} + H_{\mathrm{Har}} + H_{\mathrm{ex}} + H_{\mathrm{SOC}}. \tag{2}$$

Here H_{K} and H_{Har} correspond to the kinetic and Hartree self-consistent potential terms, respectively, H_{ex} is the self-consistent Hartree-Fock exchange term and H_{SOC} is the spin-orbit-coupling term. H_{K} exhibits complete translational and rotational symmetry and H_{Har}, comprising the crystal periodic potential $V(\boldsymbol{r})$, is invariant under all space group operations of the crystalline system under scrutiny. Both these terms are invariant under time-reversal symmetry. H_{ex} is invariant under all magnetic space group operations, that may include time-reversal as a symmetry element on its own or in combination with spatial symmetries. When the dependence on spin-1/2 degrees of freedom is explicitly taken into account, H_{ex} is invariant under double magnetic space group symmetries. H_{SOC} is time-reversal invariant, as well as invariant under double space group operations given its explicit dependence on the electron-spin degrees of freedom.

Eigensolutions of Eq. (1) can be written in Bloch form, $\psi_{n\boldsymbol{k}}(\boldsymbol{r}) = e^{i\boldsymbol{k}\cdot\boldsymbol{r}}u_{n\boldsymbol{k}}(\boldsymbol{r})$, the crystal quasi-momentum $\boldsymbol{k}$ labeling the irreducible representations of the translation subgroup and $u_{n\boldsymbol{k}}(\boldsymbol{r})$ being a cell-periodic function labeled by a band index n (encompassing both orbital and spin degrees of freedom) and fulfilling the equation $H(\boldsymbol{k})u_{n\boldsymbol{k}}(\boldsymbol{r}) = \epsilon_n(\boldsymbol{k})u_{n\boldsymbol{k}}(\boldsymbol{r})$, where in atomic units

$$H(\boldsymbol{k}) = H + 2\boldsymbol{k}\cdot\boldsymbol{p} + k^2 + \frac{1}{c^2}\boldsymbol{k}\cdot\boldsymbol{\sigma}\times\nabla V. \tag{3}$$

Here, $\boldsymbol{p} = -i\nabla$ is the momentum operator. The little group of the wave-vector $\boldsymbol{k} = \boldsymbol{k}_0$ enables the labeling of Bloch functions using its double-group irreducible (co-)representations, whose dimension d provides information on the essential band degeneracies (including spin) at $\boldsymbol{k}_0$. Then, one has access on how the eigenfunctions transform under symmetry operations even without the need of solving the Schrödinger-like equation.

The $\boldsymbol{k}\cdot\boldsymbol{p}$ method is a perturbative approach for studying the development of eigenvalues and eigensolutions of $H(\boldsymbol{k}_0 + \boldsymbol{k})$ in the vicinity of the $\boldsymbol{k}_0$ point assuming that the solution of the unperturbed Hamiltonian $H(\boldsymbol{k}_0)$ is known. The perturbing Hamiltonian H' can be deduced with use of Bloch theorem. By writing the eigensolution at wave-vector $\boldsymbol{k}_0 + \boldsymbol{k}$ as

$$\psi_{n\boldsymbol{k}_0+\boldsymbol{k}}(\boldsymbol{r}) = e^{i\boldsymbol{k}\cdot\boldsymbol{r}}\psi_{n\boldsymbol{k}_0}(\boldsymbol{r}), \tag{4}$$

one can express the perturbation of the first order in $\boldsymbol{k}$ as

$$H'(\boldsymbol{k}) \;=\; \boldsymbol{k}\cdot\left(2\boldsymbol{p}+\frac{1}{c^2}\boldsymbol{\sigma}\times\nabla V\right)\equiv\boldsymbol{k}\cdot\boldsymbol{v}, \tag{5}$$

where a generalized velocity operator $\boldsymbol{v}$, transforming as a polar rank-1 tensor, is introduced. The unperturbed states are expressed in terms of the cell-periodic part of the Bloch functions $u_{n\boldsymbol{k}_0}$ that satisfies $H(\boldsymbol{k}_0)u_{n\boldsymbol{k}_0}=\epsilon_n(\boldsymbol{k}_0)u_{n\boldsymbol{k}_0}$. Even though the explicit unperturbed solutions are not known, group theory provides information on how they transform under symmetry operations belonging to the little group of $\boldsymbol{k}_0$. One can then apply standard quasi-degenerate perturbation theory that requires the knowledge of matrix elements

$$H'_{nn'}(\boldsymbol{k}) \;=\; \langle u_{n\boldsymbol{k}_0}|\,H'(\boldsymbol{k})\,|u_{n'\boldsymbol{k}_0}\rangle=\boldsymbol{k}\cdot\langle u_{n\boldsymbol{k}_0}|\,\boldsymbol{v}\,|u_{n'\boldsymbol{k}_0}\rangle\equiv\boldsymbol{k}\cdot\langle\boldsymbol{v}\rangle_{nn'}. \tag{6}$$

Symmetry constraints on such matrix elements can be enforced using the same master equation as introduced for the expectation value of spin (see Eq. 1 in the main text):

$$\langle\boldsymbol{v}'\rangle_{nn'} \;=\; \sum_{k,k'}D_{nk}(g)D^*_{n'k'}(g)\langle\boldsymbol{v}\rangle_{kk'} \tag{7}$$

where $\boldsymbol{v}'=g\boldsymbol{v}g^{-1}$ and $D_{nk}(g)$ is a $d\times d$ matrix representation of group element g for the d-dimensional irreducible representation of the unperturbed wave-functions. Notice that unperturbed states n and n' need not to transform as the same irreducible representation. It is worth noting that spins transform as axial vectors, while (generalized) velocities transform as polar vectors, resulting in different symmetry constraints in the presence of inversion/reflection operations. Nonzero matrix elements allowed by symmetry can be deduced by applying the master equation for all elements in the group.

In general, the perturbed wave functions can be written as linear combinations of unperturbed ones, $u_{n\boldsymbol{k}_0}$, as inherently allowed by a perturbation-theory approach. This is true both for degenerate and non-degenerate bands. In the former case, the knowledge of non-zero matrix elements $\left\langle u^{\Gamma_i}_{n\boldsymbol{k}_0\alpha}\middle|\,\boldsymbol{v}\,\middle|u^{\Gamma_i}_{n\boldsymbol{k}_0\beta}\right\rangle$ is required to construct the perturbed $d\times d$ Hamiltonian $H^{\Gamma_i}(\boldsymbol{k})$, where now $\alpha,\beta=1...d$ label the α-th, β-th components of basis functions for irreducible representation Γ_i accounting for the transformation of degenerate eigensolutions under the elements of the group. Within degenerate first-order perturbation theory, the eigensolutions at small $\boldsymbol{k}$ can be generally written in the basis of unperturbed eigensolutions at $\boldsymbol{k}_0$:

$$u_{n\boldsymbol{k}\alpha} \;=\; \sum_\beta c_{\alpha\beta}\,u^{\Gamma_i}_{n\boldsymbol{k}_0\beta} \tag{8}$$

where $c_{\alpha\beta}$ are coefficients that can be determined by diagonalizing the perturbed Hamiltonian. On the other hand, for a nondegenerate manifold whose wave-function transforms as a one-dimensional irreducible representation Γ_i, the first-order perturbed wave-function can be expressed as

$$u_{n\boldsymbol{k}} \;=\; u^{\Gamma_i}_{n\boldsymbol{k}_0}+\sum_{m\neq n}\sum_\beta\frac{\left\langle u^{\Gamma_j}_{m\boldsymbol{k}_0\beta}\middle|\,H'\,\middle|u^{\Gamma_i}_{n\boldsymbol{k}_0}\right\rangle}{\epsilon_n(\boldsymbol{k}_0)-\epsilon_m(\boldsymbol{k}_0)}u^{\Gamma_j}_{m\boldsymbol{k}_0\beta} \tag{9}$$

where now the unperturbed eigensolutions with band index m can transform as any of the irreducible representations Γ_j of the little group at $\boldsymbol{k}_0$ (including Γ_i). A similar correction can be included as a higher-order correction for degenerate manifolds.

It follows that the spin texture around a given (high-symmetry) point $\boldsymbol{k}_0$ in the Brillouin zone can always be deduced by knowing the allowed spin-matrix elements at the targeted $\boldsymbol{k}_0$. In particular, if all irreducible representations at the targeted point only allow for a uniaxial component, no spin-mixing occurs that can affect the persistent uniaxial spin polarization. When different irreducible representations enforce different constraints on spin-matrix elements, instead, spin polarization around the targeted $\boldsymbol{k}_0$ may deviate from persistent uniaxial directions of an amount that is inversely proportional to energy separation of involved bands, provided that (generalized) velocity matrix elements are nonzero as enforced by the master equation Eq. (7) . Unfortunately, symmetry considerations do not allow one to estimate the range of validity of perturbation theory or the width of the area where the persistent uniaxial spin polarization is maintained, as this would require explicitly calculating unperturbed wave functions to get access to matrix elements and/or unperturbed eigenvalues to get information on energy separation of

bands. As a rule of thumb, however, for a band displaying uniaxial spin polarization at high-symmetry points/lines one can expect that the greater the energy separation from other bands, the larger the region in reciprocal space where persistent uniaxial spin polarization is realized.

Example. Let us consider the Z point of SG 4 ($P2_1$). Here, there are two possible irreducible corepresentations labeled as $\bar{Z}_3\bar{Z}_3$ and $\bar{Z}_4\bar{Z}_4$ at the Bilbao Crystallographic Server (BCS), and any band at Z point will belong to one of these two representations. We label the electronic states as $u^{\Gamma_i}_{n\alpha}$, each being two-fold degenerate and where Γ_i is one of the two irreducible representations; we also introduce a shorthand notation $|n, \Gamma_i, \alpha\rangle$. The little group of Z contains two nontrivial symmetry elements, i.e., 2 and $2'$, with matrix representations given by:

$$D(2) = \begin{pmatrix} \pm 1 & 0 \\ 0 & \pm 1 \end{pmatrix} \qquad D(2') = \begin{pmatrix} 0 & \mp 1 \\ \pm 1 & 0 \end{pmatrix}. \tag{10}$$

Here, the upper signs refer to $\Gamma_1 = \bar{Z}_3\bar{Z}_3$ and lower signs to $\Gamma_2 = \bar{Z}_4\bar{Z}_4$. The conventional setting has the rotational axis parallel to the b axis, that one can take parallel to a cartesian z direction.

Now, we have two different vector operators to consider, velocity and spin. Both are odd under time-reversal, the former being polar and the latter being axial. In this simple example, they transform in the same way, since the little group contains only rotations (no inversion, no reflection). For determining which spin polarization is allowed at the Z point, we need to apply the master equation for spin within each representation only. Applying the master equation for $g = 2$, we recover:

$$\langle \sigma_{x,y} \rangle_{i\alpha,i\beta} = 0 \qquad \langle \sigma_z \rangle_{i\alpha,i\beta} \neq 0 \tag{11}$$

where now $i = 1, 2$ denotes the representations Γ_1 or Γ_2, respectively, while $\alpha, \beta = 1, 2$ denote components of basis functions for each representation. By applying the master equation for $g = 2'$, we get additional relations between different matrix elements within both degenerate manifolds/representations

$$\langle \sigma_z \rangle_{i1,i2} = \langle \sigma_z \rangle_{i2,i1} \qquad \langle \sigma_z \rangle_{i1,i1} = -\langle \sigma_z \rangle_{i2,i2}. \tag{12}$$

Since the velocity operator here transforms as the spin, the very same constraints apply to velocity matrix elements within each degenerate subspace/representation. Then, we can introduce $\langle v_z \rangle_{i1,i2} = A_i$ and $\langle v_z \rangle_{i1,i1} = B_i$ and construct a $\boldsymbol{k} \cdot \boldsymbol{p}$ model using degenerate perturbation theory for any two-fold degenerate band $\epsilon_n^{\Gamma_i}(\boldsymbol{k}_0 = Z) \equiv \epsilon_{n,i}$ indexed by n and transforming as Γ_i representation:

$$H_{n,i}(\boldsymbol{k}) = \begin{pmatrix} \epsilon_{n,i} + B_i k_z & A_i k_z \\ A_i k_z & \epsilon_{n,i} - B_i k_z \end{pmatrix}. \tag{13}$$

One can also introduce Pauli matrices and write the model more compactly as

$$H_{n,i}(\boldsymbol{k}) = \epsilon_{n,i}\sigma_0 + B_i k_z \sigma_z + A_i k_z \sigma_x \tag{14}$$

resulting in the band dispersion close to the Z point

$$\epsilon^{\pm}_{n,i}(\boldsymbol{k}) = \epsilon_{n,i} \pm \sqrt{A_i^2 + B_i^2}\,|k_z| \tag{15}$$

with eigensolutions

$$|n, \Gamma_i, +\rangle = \cos\frac{\theta}{2}\,|n, \Gamma_i, 1\rangle + \sin\frac{\theta}{2}\,|n, \Gamma_i, 2\rangle \tag{16}$$

$$|n, \Gamma_i, -\rangle = -\sin\frac{\theta}{2}\,|n, \Gamma_i, 1\rangle + \cos\frac{\theta}{2}\,|n, \Gamma_i, 2\rangle \tag{17}$$

(18)

where $\tan\theta = A_i/B_i$. Note that we kept here the Γ_i label for perturbed wave functions as a reminder of the starting unperturbed manifold/representation. If now one needs to calculate the spin polarization, i.e., the spin expectation value close to $\boldsymbol{k}_0 = Z$, it is enough to express $\langle \pm|\boldsymbol{\sigma}|\pm\rangle$ as matrix elements on unperturbed states $|n, \Gamma_i, \alpha\rangle$, which are nonzero only for σ_z component. Specifically, one has

$$\langle \pm|\,\sigma_z\,|\pm\rangle = \pm\Big(\cos\theta\langle\sigma_z\rangle_{11} + \sin\theta\langle\sigma_z\rangle_{12}\Big). \tag{19}$$

For unperturbed degenerate manifolds, this result would be general to leading order in $\boldsymbol{k}\cdot\boldsymbol{p}$ perturbation theory, as it originates from "intraband" mixing (where only matrix elements within the degenerate subspace appear).

Now, let's consider "interband" mixing. Clearly, the interband mixing between two pairs of degenerate bands transforming as the same representation will fulfill the same symmetry constraints found above. Let us consider for instance two manifolds both transforming as Γ_1. Then, we can construct a 4×4 Hamiltonian

$$H(\boldsymbol{k}) = \begin{pmatrix} \epsilon_n + B_n k_z & A_n k_z & B_{nm} k_z & A_{nm} k_z \\ A_n k_z & \epsilon_n - B_n k_z & A_{nm} k_z & -B_{nm} k_z \\ B_{nm} k_z & A_{nm} k_z & \epsilon_m + B_m k_z & A_m k_z \\ A_{nm} k_z & -B_{nm} k_z & A_m k_z & \epsilon_m - B_m k_z \end{pmatrix} \quad (20)$$

where $A_{nm} = \langle n, \Gamma_1, 1 | \, v_z \, | m, \Gamma_1, 2 \rangle$ and $B_{nm} = \langle n, \Gamma_1, 1 | \, v_z \, | m, \Gamma_1, 1 \rangle$. One can diagonalize this Hamiltonian or recast it in block-diagonal form using quasi-degenerate perturbation theory (or Löwdin partitioning [1]). Anyhow, given that the constraints on spin matrix elements evaluated within the same representation, the uniaxial spin polarization will be kept.

If instead the two pairs of degenerate bands belong to different representations one needs to consider also matrix elements of the form $\langle n, \Gamma_1, \alpha | \, \boldsymbol{a} \, | m, \Gamma_2, \beta \rangle$, that can be deduced from the master equation considering the appropriate representation matrices, e.g.,

$$\langle n, \Gamma_1, \alpha | \, \boldsymbol{a} \, | m, \Gamma_2, \beta \rangle \, \boldsymbol{R} = \sum_{\alpha', \beta'} D^{\Gamma_1}_{\alpha\alpha'}(g) [D^{\Gamma_2}_{\beta\beta'}(g)]^* \, \langle n, \Gamma_1, \alpha' | \, \boldsymbol{a} \, | m, \Gamma_2, \beta' \rangle \quad (21)$$

Now, symmetry constraints change such that:

$$\langle a_\alpha \rangle_{11,22} = \langle a_\alpha \rangle_{12,21} \qquad \langle a_\alpha \rangle_{11,21} = -\langle a_\alpha \rangle_{12,22} \quad (22)$$

where $\alpha = x, y$. Notice that these equalities apply both to velocity and spin vector operators. This already tells us that uniaxial spin polarization will be disrupted by the admixture between bands belonging to different representations, as matrix elements of spin between states transforming as Γ_1 and Γ_2 will be nonzero for components x, y different from the uniaxial direction within each manifold. Introducing $\langle v_\alpha \rangle_{n\Gamma_1 1, m\Gamma_2 1} = C_{nm,\alpha}$ and $\langle v_\alpha \rangle_{n\Gamma_1 1, m\Gamma_2 2} = D_{nm,\alpha}$, the $\boldsymbol{k}\cdot\boldsymbol{p}$ Hamiltonian now will read:

$$H(\boldsymbol{k}) = \begin{pmatrix} \epsilon_{n,\Gamma_1} + B_{n\Gamma_1} k_z & A_{n\Gamma_1} k_z & C_{nm,x} k_x + C_{nm,y} k_y & D_{nm,x} k_x + D_{nm,y} k_y \\ A_n k_z & \epsilon_{n,\Gamma_1} - B_{n\Gamma_1} k_z & D_{nm,x} k_x + D_{nm,y} k_y & -C_{nm,x} k_x - C_{nm,y} k_y \\ C_{nm,x} k_x + C_{nm,y} k_y & D_{nm,x} k_x + D_{nm,y} k_y & \epsilon_{m,\Gamma_2} + B_{m\Gamma_2} k_z & A_{m\Gamma_2} k_z \\ D_{nm,x} k_x + D_{nm,y} k_y & -C_{nm,x} k_x - C_{nm,y} k_y & A_m k_z & \epsilon_{m,\Gamma_2} - B_{m\Gamma 2} k_z \end{pmatrix} \quad (23)$$

From here one can deduce that the bands admixture responsible for spin canting will occur only along k_x, k_y directions. Again, the Hamiltonian can be block-diagonalized using Löwdin partitioning. Notice that corrections arising from "inter-manifold" mixing will be inversely proportional to energy differences.

Supplementary note 2

Remarks on the reciprocal lattice vectors

The choice of unit cells and the naming of the points in the BZ in materials databases such as AFLOWLIB are based on the convention set by Setyawan and Curtarolo [2]. However, the COREPRESENTATIONS tool in Bilbao Crystallographic Server (BCS) defines each k-vector using the conventional basis as were listed by Cracknell, Davies, Miller and Love [3, 4, 5, 6, 7, 8], where the choice of unique axis and the definitions of unit cells are different than in the SC convention. By comparison of the little group symmetries of each k-point type in BCS with the symmetries of the corresponding BZs in [2], we determine the conventional BCS basis and represent them as k_x, k_y and k_z vectors in the BZ sketches below. The conventional basis better reflects the symmetries of the crystal, allowing us to conveniently denote the PST directions such as [100] and [010]. Different shapes of BZs for conventional lattices result from different possibilities for the lengths of the real space lattice vectors and angles. Since the COREPRESENTATIONS tool lists the representations for points belonging to only the first kind of BZ shape for each Bravais lattice type (see Fig. S1-S5), the k-points, lines, and planes in Table S1 refer to the points on the first BZ for each Bravais lattice, such as $MCLC_1$ and RHL_1.

Monoclinic (SGs 3-15)

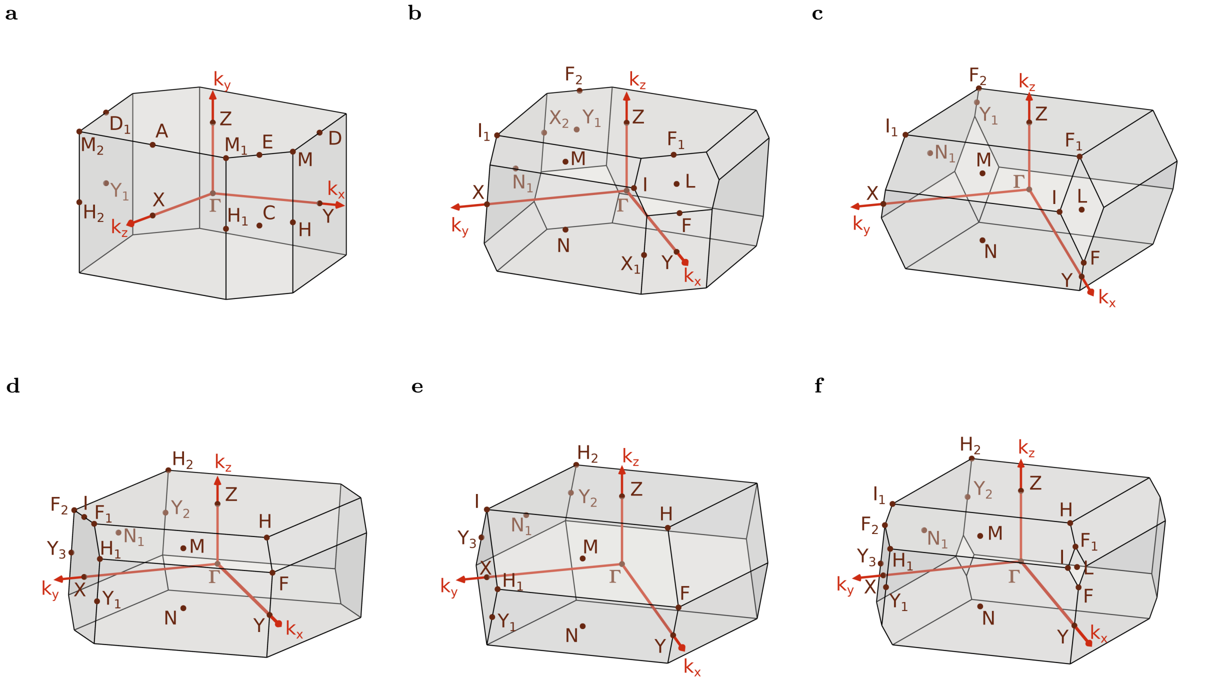

Supplementary figure 1: BZ of monoclinic (a) primitive, (b) c-centered $MCLC_1$, (c) c-centered $MCLC_2$, (d) c-centered $MCLC_3$, (e) c-centered $MCLC_4$, and (f) c-centered $MCLC_5$ lattice. The BCS basis is represented by k_x, k_y, k_z vectors. Naming of the high-symmetry points follows the SC convention [2].

Orthorhombic (SGs 16-74)

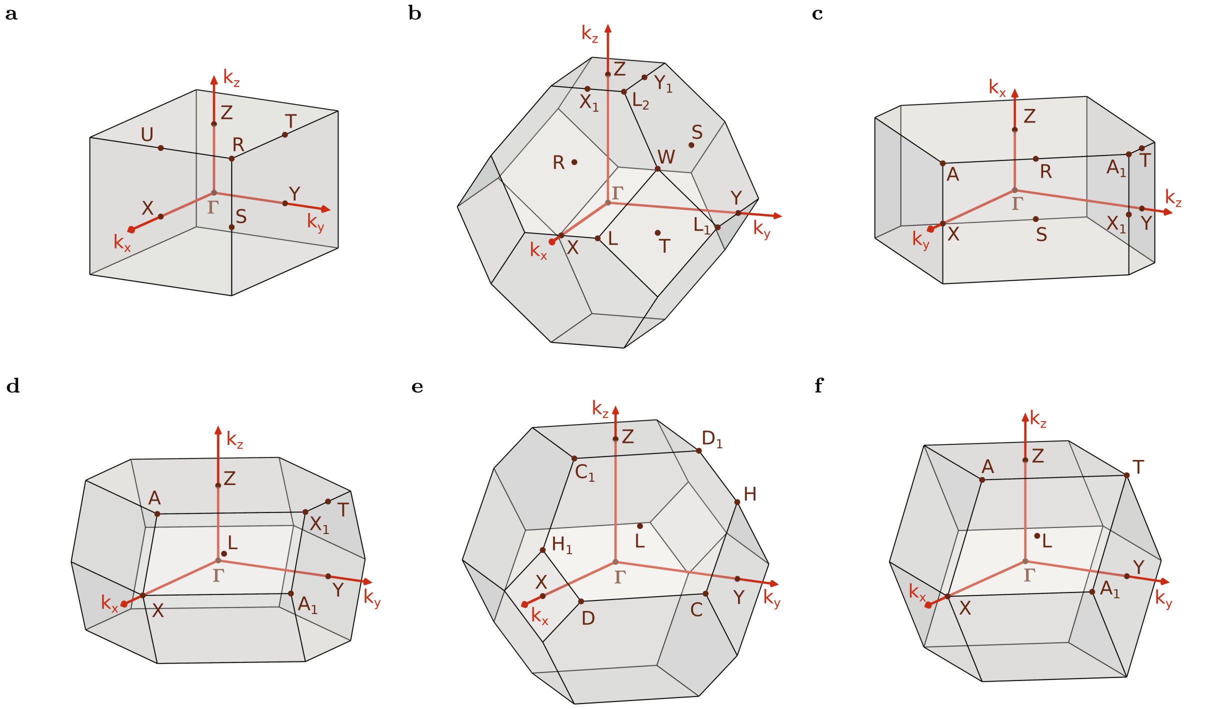

Supplementary figure 2: Same as Fig. S1 for the orthorhombic (a) primitive, (b) body-centered, (c) c-centered, (d) face-centered $ORCF_1$, (e) face-centered $ORCF_2$, and (f) face-centered $ORCF_3$ lattice.

Tetragonal (SGs 75-142)

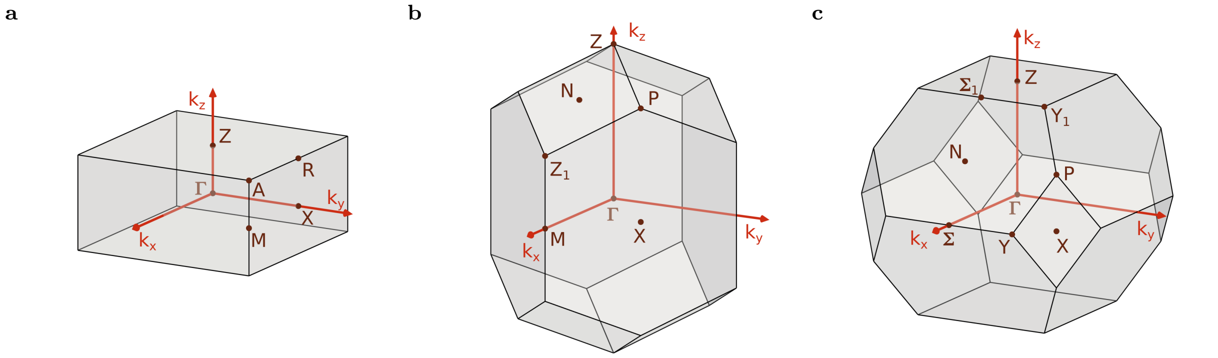

Supplementary figure 3: Same as Fig. S1 for the tetragonal (a) primitive, (b) body-centered BCT_1, and (c) body-centered BCT_2 lattice.

Trigonal and Hexagonal (SGs 143-194)

a **b** **c**

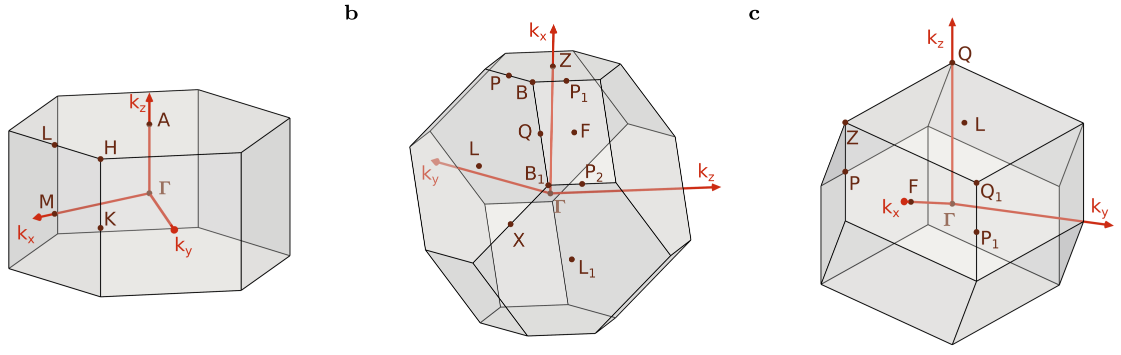

Supplementary figure 4: Same as Fig. S1 for the hexagonal (a) primitive, (b) rhombohedral RHL_1, and (c) rhombohedral RHL_2 lattice.

Cubic (SGs 195-230)

a **b** **c**

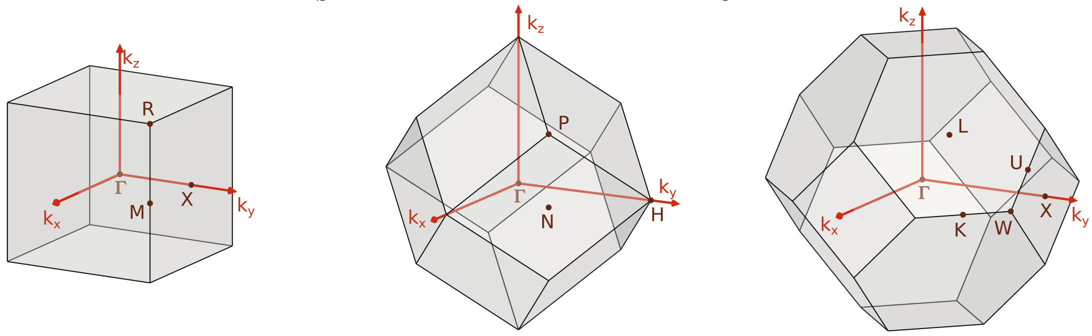

Supplementary figure 5: Same as Fig. S1 for the cubic (a) primitive, (b) body-centered, and (c) face-centered lattice.

Supplementary note 3

Additional data for Be_5Pt

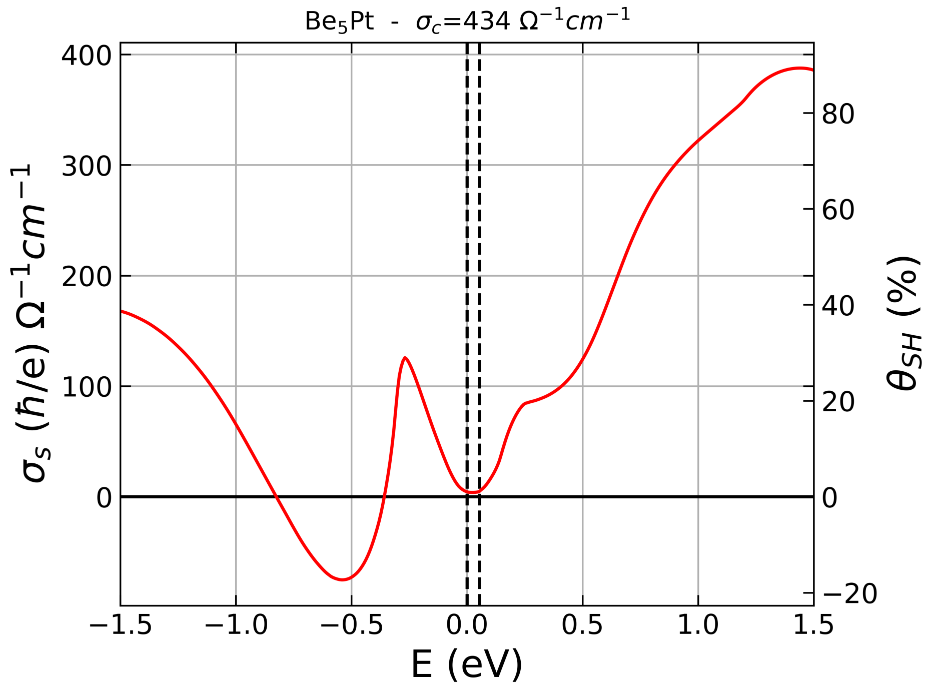

Supplementary figure 6: Calculation of the conventional spin Hall effect for Be_5Pt. The left y-axis corresponds to the calculated spin Hall conductivity, and the right y-axis indicates the corresponding spin Hall angle. The spin Hall angle was obtained using an experimental value of the charge conductivity of 434 $\Omega^{-1}\text{cm}^{-1}$ [9]. The mesh for the interpolated PAO Hamiltonian was set to $72 \times 72 \times 72$. The energy region between the dashed lines corresponds to the band gap.

Supplementary note 4

Table S1: Table of the noncentrosymmetric crystallographic space groups, including high-symmetry points, lines, and planes in the Brillouin zone (BZ) with symmetry-protected persistent spin texture (SP-PST). Note that we use the grey groups containing time reversal symmetry ($1'$) which is omitted in the space group symbols for simplicity. Normal and bold fonts denote type I and type II PST, respectively. The spin direction is indicated in square brackets and expressed in terms of the conventional reciprocal lattice vectors. The definition of the reciprocal lattice vectors and the high-symmetry k-points follows the Setyawan-Curtarolo (SC) convention [2] (see Supplementary note 2). The space groups that have an underlined number belong to the chiral (Sohncke) groups; we note that chiral groups, due to the lack of mirror symmetries, do not support PST on planes.

SG		Points	Lines	Planes
Triclinic				
1	$P1$	–	–	–
Monoclinic				
3	$P2$	–	[010] ΓZ, YD, XA, EC	–
4	$P2_1$	[010] **Z**, **E**, **D**, **A**	[010] ΓZ, YD, XA, EC	–
5	$C2$	–	[010] ΓX, ZI_1	–
6	Pm	–	–	[010] ΓYX, ZDA
7	Pc	[010] **X**, **E**, **C**, **A**	–	[010] ΓYX, ZDA
8	Cm	–	–	[010] ΓZY
9	Cc	[010] **Z**, $\mathbf{I_1}$	–	[010] ΓZY
Orthorhombic				
16	$P222$	–	[100] ΓX, ZU, YS, TR<br>[010] ΓY, ZT, XS, UR<br>[001] ΓZ, YT, XU, SR	–
17	$P222_1$	[001] **Z**, **U**, **T**, **R**	[100] ΓX, YS<br>[010] ΓY, XS<br>[001] ΓZ, YT, XU, SR	–
18	$P2_12_12$	[100] **X**, **U**<br>[010] **Y**, **T**	[100] ΓX, ZU<br>[010] ΓY, ZT<br>[001] ΓZ, **SR**	–
19	$P2_12_12_1$	[100] **X**<br>[010] **Y**<br>[001] **Z**	[100] ΓX, **TR**<br>[010] ΓY, **UR**<br>[001] ΓZ, **SR**	–
20	$C222_1$	[001] **Z**, **T**, **R**	[100] ΓY,<br>[010] ΓX<br>[001] ΓZ, YT, SR	–
21	$C222$	–	[100] ΓY, ZT<br>[010] ΓX, ZA<br>[001] ΓZ, YT, SR	–
22	$F222$	–	[100] ΓX, ZA<br>[010] ΓY, ZT<br>[001] ΓZ, YT	–
23	$I222$	–	[100] ΓX, WS<br>[010] ΓY, WR<br>[001] ΓZ, WT	–
24	$I2_12_12_1$	–	[100] ΓX, WS<br>[010] ΓY, WR<br>[001] ΓZ, WT	–
25	$Pmm2$	–	[100] ΓY, ZT, XS, UR<br>[010] ΓX, ZU, YS, TR	[100] ΓZY, XUS<br>[010] ΓZX, YTS
26	$Pmc2_1$	–	[100] ΓY, **ZT**, XS, **UR**<br>[010] ΓX, YS	[100] ΓZY, XUS<br>[010] ΓZX, YTS
27	$Pcc2$	–	[100] ΓY, XS<br>[010] ΓX, YS	[100] ΓZY, XUS<br>[010] ΓZX, YTS

Continued on next page

28	$Pma2$	[010] **X**, **U**, **S**, **R**	[100] ΓY, ZT<br>[010] ΓX, ZU, YS, TR	[100] ΓZY, XUS<br>[010] ΓZX, YTS
29	$Pca2_1$	[010] **X**, **S**	[100] ΓY, **UR**<br>[010] ΓX, **ZU**, YS, **TR**	[100] ΓZY, XUS<br>[010] ΓZX, YTS
30	$Pnc2$	[100] **Y**, **S**<br>[010] **T**, **R**	[100] ΓY, XS<br>[010] ΓX, TR	[100] ΓZY, XUS<br>[010] ΓZX, YTS
31	$Pmn2_1$	[010] **X**, **S**<br>[001] **U**, **R**	[100] ΓY, **ZT**<br>[010] ΓX, YS	[100] ΓZY, XUS<br>[010] ΓZX, YTS
32	$Pba2$	[100] **Y**, **T**<br>[010] **X**, **U**	[100] ΓY, ZT<br>[010] ΓX, ZU	[100] ΓZY, XUS<br>[010] ΓZX, YTS
33	$Pna2_1$	[100] **Y**<br>[010] **X**<br>[001] **T**	[100] ΓY, **UR**<br>[010] ΓX, **ZU**	[100] ΓZY, XUS<br>[010] ΓZX, YTS
34	$Pnn2$	[100] **Y**, **U**<br>[010] **X**, **T**	[100] ΓY, UR<br>[010] ΓX, TR	[100] ΓZY, XUS<br>[010] ΓZX, YTS
35	$Cmm2$	–	[100] ΓX, ZA<br>[010] ΓY, ZT<br>[001] SR	[100] ΓZY<br>[010] ΓZX
36	$Cmc2_1$	[001] **R**	[100] ΓX, **ZA**<br>[010] ΓY<br>[001] SR	[100] ΓZX<br>[010] ΓZY
37	$Ccc2$	–	[100] ΓX<br>[010] ΓY<br>[001] SR	[100] ΓZX<br>[010] ΓZY
38	$Amm2$	–	[100] ΓX, ZA<br>[010] ΓZ, YT	[100] ΓYX, ZTA<br>[010] ΓZY
39	$Aem2$	[100] **S**, **R**	[100] ΓX, ZA<br>[010] ΓZ, YT	[100] ΓYX, ZTA<br>[010] ΓZY
40	$Ama2$	[010] **Z**, **T**	[100] ΓX<br>[010] ΓZ, YT	[100] ΓYX, ZTA<br>[010] ΓZY
41	$Aea2$	[100] **S**, **R**<br>[010] **Z**, **T**	[100] ΓX<br>[010] ΓZ, YT	[100] ΓYX, ZTA<br>[010] ΓZY
42	$Fmm2$	–	[100] ΓY, ZT<br>[010] ΓX, ZA	[100] ΓZY<br>[010] ΓZX
43	$Fdd2$	[100] **Y**<br>[010] **X**	[100] ΓY<br>[010] ΓX	[100] ΓZY<br>[010] ΓZX
44	$Imm2$	[001] W	[100] ΓY<br>[010] ΓX<br>[001] WT	[100] ΓZY<br>[010] ΓZX
45	$Iba2$	[100] **S**<br>[010] **R**<br>[001] **W**	[100] ΓY<br>[010] ΓX<br>[001] WT	[100] ΓZY<br>[010] ΓZX
46	$Ima2$	[010] **R**	[100] ΓY<br>[010] ΓX<br>[001] WT	[100] ΓZY<br>[010] ΓZX
Tetragonal				
75	$P4$	–	[001] ΓZ, XR, MA	–
76	$P4_1$	[001] **Z**, **R**, **A**	[001] ΓZ, XR, MA	–
77	$P4_2$	–	[001] ΓZ, XR, MA	–
78	$P4_3$	[001] **Z**, **R**, **A**	[001] ΓZ, XR, MA	–
79	$I4$	–	[001] ΓZ, XP	–
80	$I4_1$	[001] **P****	[001] ΓZ, XP	–
81	$P\bar{4}$	–	[001] XR	–
82	$I\bar{4}$	[001] P	[001] XP	–

Continued on next page

89	$P422$	–	[100] XM, RA<br>[010] ΓX, ZR<br>[001] ΓZ, XR, MA<br>[110] ΓM, ZA	–
90	$P42_12$	[010] $\mathbf{X}$, $\mathbf{R}$	[010] ΓX, ZR<br>[001] ΓZ, $\mathbf{MA}$<br>[110] ΓM, ZA	–
91	$P4_122$	[001] $\mathbf{Z}^*$, $\mathbf{R}$, $\mathbf{A}^*$	[100] XM<br>[010] ΓX<br>[001] ΓZ, XR, MA<br>[110] ΓM	–
92	$P4_12_12$	[010] $\mathbf{X}$<br>[001] $\mathbf{Z}^*$, $\mathbf{A}^*$	[100] $\mathbf{RA}$<br>[010] ΓX<br>[001] ΓZ, $\mathbf{MA}$<br>[110] ΓM	–
93	$P4_222$	–	[100] XM, RA<br>[010] ΓX, ZR<br>[001] ΓZ, XR, MA<br>[110] ΓM, ZA	–
94	$P4_22_12$	[010] $\mathbf{X}$, $\mathbf{R}$	[010] ΓX, ZR<br>[001] ΓZ, $\mathbf{MA}$<br>[110] ΓM, ZA	–
95	$P4_322$	[001] $\mathbf{Z}^*$, $\mathbf{R}$, $\mathbf{A}^*$	[100] XM<br>[010] ΓX<br>[001] ΓZ, XR, MA<br>[110] ΓM	–
96	$P4_32_12$	[010] $\mathbf{X}$<br>[001] $\mathbf{Z}^*$, $\mathbf{A}^*$	[100] $\mathbf{RA}$<br>[010] ΓX<br>[001] ΓZ, $\mathbf{MA}$<br>[110] ΓM	–
97	$I422$	–	[100] ΓM<br>[010] PN<br>[001] ΓZ, XP<br>[110] ΓX<br>[1$\bar{1}$0] XM	–
98	$I4_122$	[001] $\mathbf{P}^*$	[100] ΓM<br>[010] PN<br>[001] ΓZ, XP<br>[110] ΓX<br>[1$\bar{1}$0] XM	–
99	$P4mm$	–	[100] ΓX, ZR<br>[010] XM, RA<br>[1$\bar{1}$0] ΓM, ZA	[100] ΓZX<br>[010] XRM<br>[1$\bar{1}$0] ΓZM
100	$P4bm$	[100] $\mathbf{X}$, $\mathbf{R}$	[100] ΓX, ZR<br>[1$\bar{1}$0] ΓM, ZA	[100] ΓZX<br>[010] XRM<br>[1$\bar{1}$0] ΓZM
101	$P4_2cm$	–	[100] ΓX<br>[010] XM<br>[1$\bar{1}$0] ΓM, ZA	[100] ΓZX<br>[010] XRM<br>[1$\bar{1}$0] ΓZM
102	$P4_2nm$	[100] $\mathbf{X}$<br>[010] $\mathbf{R}$	[100] ΓX<br>[010] RA<br>[1$\bar{1}$0] ΓM, ZA	[100] ΓZX<br>[010] XRM<br>[1$\bar{1}$0] ΓZM
103	$P4cc$	–	[100] ΓX<br>[010] XM<br>[1$\bar{1}$0] ΓM	[100] ΓZX<br>[010] XRM<br>[1$\bar{1}$0] ΓZM
104	$P4nc$	[100] $\mathbf{X}$<br>[010] $\mathbf{R}$	[100] ΓX<br>[010] RA<br>[1$\bar{1}$0] ΓM	[100] ΓZX<br>[010] XRM<br>[1$\bar{1}$0] ΓZM

Continued on next page

105	$P4_2mc$	–	[100] ΓX, ZR<br>[010] XM, RA<br>$[1\bar{1}0]$ ΓM	[100] ΓZX<br>[010] XRM<br>$[1\bar{1}0]$ ΓZM
106	$P4_2bc$	[100] **X**, **R**	[100] ΓX, ZR<br>$[1\bar{1}0]$ ΓM	[100] ΓZX<br>[010] XRM<br>$[1\bar{1}0]$ ΓZM
107	$I4mm$	–	[010] ΓM<br>[110] XM<br>$[1\bar{1}0]$ ΓX	[010] ΓZM<br>$[1\bar{1}0]$ ΓZX
108	$I4cm$	[010] **N**	[010] ΓM<br>[110] XM<br>$[1\bar{1}0]$ ΓX	[010] ΓZM<br>$[1\bar{1}0]$ ΓZX
109	$I4_1md$	[001] $\mathbf{P}^*$<br>$[1\bar{1}0]$ **X**	[010] ΓM<br>$[1\bar{1}0]$ ΓX	[010] ΓZM<br>$[1\bar{1}0]$ ΓZX
110	$I4_1cd$	[010] **N**<br>[001] $\mathbf{P}^*$<br>$[1\bar{1}0]$ **X**	[010] ΓM<br>$[1\bar{1}0]$ ΓX	[010] ΓZM<br>$[1\bar{1}0]$ ΓZX
111	$P\bar{4}2m$	–	[100] XM, RA<br>[010] ΓX, ZR<br>[001] XR<br>$[1\bar{1}0]$ ΓM, ZA	$[1\bar{1}0]$ ΓZM
112	$P\bar{4}2c$	–	[100] XM, RA<br>[010] ΓX, ZR<br>[001] XR<br>$[1\bar{1}0]$ ΓM	$[1\bar{1}0]$ ΓZM
113	$P\bar{4}2_1m$	[010] **X**, **R**	[010] ΓX, ZR<br>$[1\bar{1}0]$ ΓM, ZA	$[1\bar{1}0]$ ΓZM
114	$P\bar{4}2_1c$	[010] **X**, **R**	[010] ΓX, ZR<br>$[1\bar{1}0]$ ΓM	$[1\bar{1}0]$ ΓZM
115	$P\bar{4}m2$	–	[100] ΓX, ZR<br>[010] XM, RA<br>[110] ΓM, ZA	[100] ΓZX<br>[010] XRM
116	$P\bar{4}c2$	–	[100] ΓX<br>[010] XM<br>[110] ΓM, ZA	[100] ΓZX<br>[010] XRM
117	$P\bar{4}b2$	[100] **X**, **R**	[100] ΓX, ZR<br>[110] ΓM, ZA	[100] ΓZX<br>[010] XRM
118	$P\bar{4}n2$	[100] **X**<br>[010] **R**	[100] ΓX<br>[010] RA<br>[110] ΓM, ZA	[100] ΓZX<br>[010] XRM
119	$I\bar{4}m2$	[001] P	[010] ΓM<br>[001] XP<br>[110] ΓX<br>$[1\bar{1}0]$ XM	[010] ΓZM
120	$I\bar{4}c2$	[010] **N**<br>[001] **P**	[010] ΓM<br>[001] XP<br>[110] ΓX<br>$[1\bar{1}0]$ XM	[010] ΓZM
121	$I\bar{4}2m$	–	[100] ΓM<br>[010] PN<br>[110] XM<br>$[1\bar{1}0]$ ΓX	$[1\bar{1}0]$ ΓZX
122	$I\bar{4}2d$	[001] $\mathbf{P}^*$<br>$[1\bar{1}0]$ **X**	[100] ΓM<br>[010] PN<br>$[1\bar{1}0]$ ΓX	$[1\bar{1}0]$ ΓZX
Trigonal				
143	$P3$	[001] $\mathbf{\Gamma}^*$, K, H, $\mathbf{A}^*$	[001] ΓA, KH	–
144	$P3_1$	[001] $\mathbf{\Gamma}^*$, K, H, $\mathbf{A}^*$	[001] ΓA, KH	–

Continued on next page

145	$P3_2$	[001] $\mathbf{\Gamma}^*, K, H, \mathbf{A}^*$	[001] $\Gamma A, KH$	–
146	$R3$	[001] $\mathbf{\Gamma}^*, \mathbf{Z}^*$	[001] ΓZ	–
149	$P312$	[001] $\mathbf{\Gamma}^*, K, H, \mathbf{A}^*$	[001] $\Gamma A, KH$<br>[210] $\Gamma M, LA$	–
150	$P321$	[001] $\mathbf{\Gamma}^*, \mathbf{A}^*$	[001] $\Gamma A, KH$<br>[110] $\Gamma K, HA$	–
151	$P3_112$	[001] $\mathbf{\Gamma}^*, K, H, \mathbf{A}^*$	[001] $\Gamma A, KH$<br>[210] $\Gamma M, LA$	–
152	$P3_121$	[001] $\mathbf{\Gamma}^*, \mathbf{A}^*$	[001] $\Gamma A, KH$<br>[110] $\Gamma K, HA$	–
153	$P3_212$	[001] $\mathbf{\Gamma}^*, K, H, \mathbf{A}^*$	[001] $\Gamma A, KH$<br>[210] $\Gamma M, LA$	–
154	$P3_221$	[001] $\mathbf{\Gamma}^*, \mathbf{A}^*$	[001] $\Gamma A, KH$<br>[110] $\Gamma K, HA$	–
155	$R32$	[001] $\mathbf{\Gamma}^*, \mathbf{Z}^*$	[010] ΓX<br>[001] ΓZ<br>[110] ZB	–
156	$P3m1$	[001] $\mathbf{\Gamma}^*, K, H, \mathbf{A}^*$	[010] $\Gamma M, ML, LA$<br>[001] KH	[010] ΓMA
157	$P31m$	[001] $\mathbf{\Gamma}^*, \mathbf{A}^*$	[1$\bar{1}$0] $\Gamma K, HA$<br>[210] ML	[1$\bar{1}$0] ΓKA
158	$P3c1$	[010] $\mathbf{L}$<br>[001] $\mathbf{\Gamma}^*, K, \mathbf{H}$	[010] $\Gamma M, ML, LA$<br>[001] KH	[010] ΓMA
159	$P31c$	[001] $\mathbf{\Gamma}^*$<br>[210] $\mathbf{L}$	[1$\bar{1}$0] $\Gamma K, HA$<br>[210] ML	[1$\bar{1}$0] ΓKA
160	$R3m$	[001] $\mathbf{\Gamma}^*, \mathbf{Z}^*$	–	[110] ΓZL
161	$R3c$	[001] $\mathbf{\Gamma}^*$<br>[110] $\mathbf{L}$	–	[110] ΓZL
Hexagonal				
168	$P6$	[001] $\mathbf{\Gamma}^*, K^*, H^*, \mathbf{A}^*$	[001] $\Gamma A, ML, KH$	–
169	$P6_1$	[001] $\mathbf{\Gamma}^*, \mathbf{L}, K^*, \mathbf{H}^*, \mathbf{A}$	[001] $\Gamma A, ML, KH$	–
170	$P6_5$	[001] $\mathbf{\Gamma}^*, \mathbf{L}, K^*, \mathbf{H}^*, \mathbf{A}$	[001] $\Gamma A, ML, KH$	–
171	$P6_2$	[001] $\mathbf{\Gamma}^*, K^*, H^*, \mathbf{A}^*$	[001] $\Gamma A, ML, KH$	–
172	$P6_4$	[001] $\mathbf{\Gamma}^*, K^*, H^*, \mathbf{A}^*$	[001] $\Gamma A, ML, KH$	–
173	$P6_3$	[001] $\mathbf{\Gamma}^*, \mathbf{L}, K^*, \mathbf{H}^*, \mathbf{A}$	[001] $\Gamma A, ML, KH$	–
174	$P\bar{6}$	[001] $\mathbf{\Gamma}^*, K, H, \mathbf{A}^*$	[001] $\Gamma M, \Gamma K, \Gamma A^*,$ LA, KH, HA	[001] $\Gamma MK, LHA$
177	$P622$	[001] $\mathbf{\Gamma}^*, \mathbf{A}^*$	[001] $\Gamma A, ML, KH$<br>[110] $\Gamma K, HA$<br>[210] $\Gamma M, LA$	–
178	$P6_122$	[001] $\mathbf{\Gamma}^*, \mathbf{L}, \mathbf{H}^*, \mathbf{A}^*$	[001] $\Gamma A, ML, KH$<br>[110] ΓK<br>[210] ΓM	–
179	$P6_522$	[001] $\mathbf{\Gamma}^*, \mathbf{L}, \mathbf{H}^*, \mathbf{A}^*$	[001] $\Gamma A, ML, KH$<br>[110] ΓK<br>[210] ΓM	–
180	$P6_222$	[001] $\mathbf{\Gamma}^*, \mathbf{A}^*$	[001] $\Gamma A, ML, KH$<br>[110] $\Gamma K, HA$<br>[210] $\Gamma M, LA$	–
181	$P6_422$	[001] $\mathbf{\Gamma}^*, \mathbf{A}^*$	[001] $\Gamma A, ML, KH$<br>[110] $\Gamma K, HA$<br>[210] $\Gamma M, LA$	–
182	$P6_322$	[001] $\mathbf{\Gamma}^*, \mathbf{L}, \mathbf{H}^*, \mathbf{A}$	[001] $\Gamma A, ML, KH$<br>[110] ΓK<br>[210] ΓM	–

Continued on next page

183	$P6mm$	[001] $\mathbf{\Gamma}^*$, $\mathbf{A}^*$	[010] ΓM, LA<br>[001] $\mathbf{\Gamma A}^*$<br>$[1\bar{1}0]$ ΓK, HA	[010] ΓMA<br>$[1\bar{1}0]$ ΓKA
184	$P6cc$	[001] $\mathbf{\Gamma}^*$, $\mathbf{H}^*$, $\mathbf{A}^*$	[010] ΓM<br>[001] $\mathbf{\Gamma A}^*$<br>$[1\bar{1}0]$ ΓK	[010] ΓMA<br>$[1\bar{1}0]$ ΓKA
185	$P6_3cm$	[001] $\mathbf{\Gamma}^*$, $\mathbf{A}^*$	[010] ΓM<br>[001] $\mathbf{\Gamma A}^*$<br>$[1\bar{1}0]$ ΓK, **HA**	[010] ΓMA<br>$[1\bar{1}0]$ ΓKA
186	$P6_3mc$	[001] $\mathbf{\Gamma}^*$, $\mathbf{H}^*$, $\mathbf{A}^*$	[010] ΓM, **LA**<br>[001] $\mathbf{\Gamma A}^*$<br>$[1\bar{1}0]$ ΓK	[010] ΓMA<br>$[1\bar{1}0]$ ΓKA
187	$P\bar{6}m2$	[001] $\mathbf{\Gamma}^*$, K, H, $\mathbf{A}^*$	[010] ML<br>[001] ΓK, KH, HA	[010] ΓMA<br>[001] ΓMK, LHA
188	$P\bar{6}c2$	[010] **L**<br>[001] $\mathbf{\Gamma}^*$, K, **H**	[010] ML<br>[001] ΓK, KH	[010] ΓMA<br>[001] ΓMK, LHA
189	$P\bar{6}2m$	[001] $\mathbf{\Gamma}^*$, $\mathbf{K}^*$, $\mathbf{H}^*$, $\mathbf{A}^*$	[001] ΓM, LA<br>[210] ML	[001] ΓMK, LHA<br>$[1\bar{1}0]$ ΓKA
190	$P\bar{6}2c$	[001] $\mathbf{\Gamma}^*$, $\mathbf{K}^*$, $\mathbf{H}^*$<br>[210] **L**	[001] ΓM<br>[210] ML	[001] ΓMK, LHA<br>$[1\bar{1}0]$ ΓKA
Cubic				
195	$P23$	–	[100] XM<br>[010] ΓX<br>[001] RM<br>[111] ΓR	–
196	$F23$	[100] W<br>[111] $\mathbf{L}^*$	[100] XW<br>[010] ΓX<br>[111] ΓL	–
197	$I23$	–	[010] ΓH<br>[001] PN<br>[111] ΓP	–
198	$P2_13$	[010] **X**	[010] ΓX<br>[001] **RM**<br>[111] ΓR	–
199	$I2_13$	–	[010] ΓH<br>[001] PN<br>[111] ΓP	–
207	$P432$	–	[100] XM<br>[010] ΓX<br>[001] RM<br>[110] ΓM<br>[101] XR<br>[111] ΓR	–
208	$P4_232$	–	[100] XM<br>[010] ΓX<br>[001] RM<br>[110] ΓM<br>[101] XR<br>[111] ΓR	–
209	$F432$	[111] $\mathbf{L}^*$	[100] XW<br>[010] ΓX<br>[110] ΓK<br>$[01\bar{1}]$ WL<br>[111] ΓL	–
210	$F4_132$	[100] $\mathbf{W}^*$<br>[111] $\mathbf{L}^*$	[100] XW<br>[010] ΓX<br>[110] ΓK<br>$[01\bar{1}]$ WL<br>[111] ΓL	–

Continued on next page

211	$I432$	–	[010] ΓH<br>[001] PN<br>[110] ΓN<br>[111] ΓP	–
212	$P4_332$	[010] **X**	[010] ΓX<br>[001] **RM**<br>[110] ΓM<br>[111] ΓR	–
213	$P4_132$	[010] **X**	[010] ΓX<br>[001] **RM**<br>[110] ΓM<br>[111] ΓR	–
214	$I4_132$	–	[010] ΓH<br>[001] PN<br>[110] ΓN<br>[111] ΓP	–
215	$P\bar{4}3m$	–	[100] XM<br>[1$\bar{1}$0] ΓM<br>[$\bar{1}$01] XR	[1$\bar{1}$0] ΓRM
216	$F\bar{4}3m$	[100] W<br>[111] **L***	[100] XW<br>[1$\bar{1}$0] ΓK	[1$\bar{1}$0] ΓLK
217	$I\bar{4}3m$	–	[1$\bar{1}$0] ΓN	[1$\bar{1}$0] ΓPN
218	$P\bar{4}3n$	–	[100] XM<br>[1$\bar{1}$0] ΓM	[1$\bar{1}$0] ΓRM
219	$F\bar{4}3c$	[100] **W**	[100] XW<br>[1$\bar{1}$0] ΓK	[1$\bar{1}$0] ΓLK
220	$I\bar{4}3d$	[1$\bar{1}$0] **N**	[1$\bar{1}$0] ΓN	[1$\bar{1}$0] ΓPN

* Type I or type II (bold) PST in some representations, and no PST for the other representations
** Type I PST for one representation and type II PST for the other representation

References

[1] Löwdin, P.-O. A note on the quantum-mechanical perturbation theory. *J. Chem. Phys.* **19**, 1396–1401 (1951).

[2] Setyawan, W. & Curtarolo, S. High-throughput electronic band structure calculations: Challenges and tools. *Comput. Mater. Sci.* **49**, 299–312 (2010).

[3] Cracknell, A., Davies, B., Miller, S. & Love, W. *Kronecker Product Tables. vol. 1. General Introduction and Tables of Irreducible Representations of Space Groups* (New York: IFI/Plenum, 1979).

[4] Bradley, C. & Cracknell, A. *The Mathematical Theory of Symmetry in Solids: Representation Theory for Point Groups and Space Groups* (Oxford University Press, 2010).

[5] Elcoro, L. *et al.* Double crystallographic groups and their representations on the *Bilbao Crystallographic Server*. *J. Appl. Cryst.* **50**, 1457–1477 (2017).

[6] Xu, Y. *et al.* High-throughput calculations of magnetic topological materials. *Nature* **586**, 702–707 (2020).

[7] Aroyo, M. I. *et al.* Brillouin-zone database on the *bilbao crystallographic server*. *Acta Crystallogr. A* **70**, 126–137 (2014).

[8] Elcoro, L. *et al.* Magnetic topological quantum chemistry. *Nat. Commun.* **12**, 5965 (2021).

[9] Fanfarillo, L. *et al.* Remarkable low-energy properties of the pseudogapped semimetal Be_5Pt. *Phys. Rev. B* **102**, 155206 (2020).
